# Tools, frameworks and resources to guide global action on strengthening rural health systems: a mapping review

**DOI:** 10.1186/s12961-023-01078-3

**Published:** 2023-12-04

**Authors:** Dewi Retno Pamungkas, Belinda O’Sullivan, Matthew McGrail, Bruce Chater

**Affiliations:** 1 Mayne Academy of Rural and Remote Medicine, Rural and Remote Medicine Clinical Unit, Medical School, Faculty of Medicine, The University of Queensland, Theodore, QLD Australia; 2 Toowoomba Regional Clinical Unit, Rural Clinical School, Faculty of Medicine, The University of Queensland, Toowoomba, QLD Australia; 3 Murray Primary Health Network, Bendigo, VIC Australia; 4 Rockhampton Regional Clinical Unit, Rural Clinical School, Faculty of Medicine, The University of Queensland, Rockhampton, QLD Australia

**Keywords:** Rural health, Health system, Frameworks, Tools, Resources, Health services, Health workforce, Rural communities

## Abstract

**Background:**

Inequities of health outcomes persist in rural populations globally. This is strongly associated with there being less health coverage in rural and underserviced areas. Increasing health care coverage in rural area requires rural health system strengthening, which subsequently necessitates having tools to guide action.

**Objective:**

This mapping review aimed to describe the range of tools, frameworks and resources (hereafter called tools) available globally for rural health system capacity building.

**Methods:**

This study collected peer-reviewed materials published in 15-year period (2005–2020). A systematic mapping review process identified 149 articles for inclusion, related to 144 tools that had been developed, implemented, and/or evaluated (some tools reported over multiple articles) which were mapped against the World Health Organization’s (WHO’s) six health system building blocks (agreed as the elements that need to be addressed to strengthen health systems).

**Results:**

The majority of tools were from high- and middle-income countries (*n* = 85, 59% and *n* = 43, 29%, respectively), and only 17 tools (12%) from low-income countries. Most tools related to the health service building block (*n* = 57, 39%), or workforce (*n* = 33, 23%). There were a few tools related to information and leadership and governance (*n* = 8, 5% each). Very few tools related to infrastructure (*n *= 3, 2%) and financing (*n* = 4, 3%). This mapping review also provided broad quality appraisal, showing that the majority of the tools had been evaluated or validated, or both (*n* = 106, 74%).

**Conclusion:**

This mapping review provides evidence that there is a breadth of tools available for health system strengthening globally along with some gaps where no tools were identified for specific health system building blocks. Furthermore, most tools were developed and applied in HIC/MIC and it is important to consider factors that influence their utility in LMIC settings. It may be important to develop new tools related to infrastructure and financing. Tools that have been positively evaluated should be made available to all rural communities, to ensure comprehensive global action on rural health system strengthening.

**Supplementary Information:**

The online version contains supplementary material available at 10.1186/s12961-023-01078-3.

## Background

Rural populations continue to experience poorer health outcomes compared to metropolitan areas [1, 2]. This difference is associated with a lack of health care coverage for 56% of rural residents, compared to only 22% in the metropolitan areas [3]. Globally, this situation includes low and middle-income countries (LMICs) and high-income countries (HICs) alike with some variation between countries and World health Organisation (WHO) regions. Improving rural health care and health outcomes relies on system strengthening at multiple levels. Drawing from the WHO system framework, such development should address the following six health system building blocks of health services, workforce, leadership and governance, information, infrastructure, and financing [4]. These are further described and defined in Box 1.

### Box 1. Definitions of frameworks/tools/resources and components of health systems

**Table:** 

**Definitions** *Frameworks, tools and resources* This mapping review applied a working definition of tools and resources as materials that inform how rural health systems can be planned and adapted to decide something. They consolidate dynamic systems information into a single point of reference to support “how to” practical action for rural health systems development. Acknowledges the many parts and their interactions needed across the system or particular system components under study (defined below). May relate a succinct diagrammatic or tabular representation of systems parts, depicting actions, processes and outcomes. May include reference to focus disease areas that could inform the wider system. *Heath systems building blocks* [4]: • *Services: *approaches to continuity of care, service integration, models of care. Services may include preventative and primary, secondary or tertiary level services • *Workforce: *training, quality improvement for performance, recruitment and retention • *Information: *electronic record keeping, data systems and information gathering • *Leadership and governance: *decentralisation, community participation, workforce licensure, accreditation and registration • *Infrastructure: *supply of materials and equipment, system architecture for integrated delivery of interventions • *Financing: *remuneration, insurance, incentives	

To support action on the health system building blocks, the WHO has focused strongly on some areas such as providing global policy recommendations to inform strategies that are most effective at supporting access to health workers through retention (released in 2010 and refreshed in 2020) [5, 6]. The WHO also endorses the use of systems thinking for considering the complex interactions within and between the various building blocks of the rural health system [4]. Systems thinking aims to demonstrate the fundamental characteristics and relationships of systems, requiring the adoption of five skills of “Dynamic thinking”, “Systems-as-cause thinking”, “Forest thinking”, “Operational thinking” and “Loop thinking” [4], p. 43]. The WHO has also developed many different guidelines focused on health service enhancement for particular disease areas such as prevention, early detection, diagnosis, treatment, or rehabilitation of infectious diseases (e.g., tuberculosis, HIV, hepatitis and malaria), though many are not specific to rural areas [7]. However, beyond the policy guidelines, rural health systems strengthening action is problematic unless rural communities and policy-makers alike have tools, resources and frameworks (hereafter called tools,, see definition in Box 1) to support planning and adaptation and decisions about implementation. Having these tools is important because they might assist the wide range of rural communities worldwide, with more standardised, evidence-based action, despite their different contexts. They can also support people with varied levels of training to understand and frame solutions together. However, there are no known collections of tools for health system strengthening that could be a reference point for policy-makers and rural communities. It is possible that there are useful tools that are applicable to rural settings that many rural communities are not aware of whilst this situation persists. Hence, this study aimed to map currently available tools to inform their applicability for rural health system strengthening.

Beyond the global community, many governments in the world have implemented strategies related to aspects of rural health system issues. This includes recent development of a national rural generalist pathway in Australia [8]. However, often the focus of systems development is in one area without consideration of the other health system building blocks, thus likely weakening their impact. An example may be focussing on rural workforce training and development without addressing the sustainability, through financing and governance of rural health service models. Having a repository of tools across the suite of health systems issues, which can be applied at all levels of health system strengthening is likely to be more effective. This could guide more holistic action and be more effective at achieving progress in rural health.

It is unclear how many rural health system strengthening tools exist, but also whether these have been co-developed with rural communities, sufficiently translated and refined, or whether they have applied consistent definitions and terms [9]. Until a thorough appraisal is done, then the range of tools and resources available for rural community action may be limited in their generalisability, lacking clarity of their broader application and difficultly in both implementing and interpreting at the global scale.

Across High to Low Income countries and their individual rural communities there are likely to be very different contexts for health system strengthening. However, having a collection of relevant tools may be helpful especially where they are developed and reviewed with consideration of their application to diverse settings and systems issues (generalisability built into the design of the tool). This includes considerations for tools to have been developed for application across High to low- income countries, different world regions including those that are extremely under-resourced and to cover different rural service types, health professions and financing contexts. The WHO recognised this when it sponsored a Rural Pathways Checklist to support the implementation of grow your own workforce strategies in low- and middle-income countries [9]. It was developed with widespread input from rural communities globally, sensitive to the Low- and Middle-Income Country resource levels, covering any rural context, all health worker types and community needs. Field-testing identified the tool was useful and applicable to High- and Middle-Income countries alike. Similar tools may be available but there has yet to be a focus by the WHO and governments, on bringing these together and sharing them with rural communities. If this occurred, it is possible for rural communities to use these tools to shape local action whilst in parallel governments can use these tools to shape policy development, thus in concert supporting coordinated action and greater efficiency. Where tools are not prescriptive, but rather enable tailoring of health systems solutions to the factors in the local context, these are likely to be important for achieving sustainable outcomes and better engagement of the rural people, who are directly affected by the decisions of local health services and policy-makers.

With this background in mind, the overall aim of this research was to review and describe the range of tools, frameworks and resources available globally to inform action on rural health system capacity building.

## Materials and methods

A mapping review was conducted because it was considered the most appropriate method for seeking out and categorising existing literature from which to commission further reviews and/or primary research [10]. Three steps were followed to identify and categorise tools, which were denoted as defined in Box 1. These aimed to encompass all components of the WHO health system building blocks [4].

### Step 1: collecting material

The search strategy was developed iteratively by two experienced rural health researchers with a background in this area and informed through further discussion of the wider research team. The search encompassed four concepts and various search terms, inclusive of rural health tool and system components (Table 1). The researchers further refined the sensitivity of the search after observing the varying interpretation and use of terms to define rural community resources. They used several peer reviewed published tools, frameworks or resources that they knew of, such as the Community Apgar to test that concepts 3 and 4 were effective in capturing relevant material. This occurred over a two-month period.

**Table 1:** Concepts used in the literature search

Concept 1	Concept 2	Concept 3	Concept 4
Rural OR “Remote” AND	Health AND	Tool OR Framework OR Model OR Handbook OR Guide OR Checklist AND	Needs assessment OR train* OR curricul* OR course OR placement OR immersion OR skill OR education OR qualification OR competen* OR recognition OR recruit* OR retention OR worker OR staffing OR service OR infrastructure OR financing OR funding OR resources OR evaluation OR monitoring

Six databases were searched, based on scope and relevance of literature content: Medline, Social Science Citation Index, CINAHL, ERIC, Rural and Remote Health, Informit Health Collection, and the Cochrane Database of Systematic reviews. All countries were included. Only articles from peer-reviewed journals and theses held in library databases were searched, thus excluding grey literature and non-published material. The reason for this was to concentrate on tools which had undergone peer review, as a form of quality control. The extent of the search was limited to March 2005–March 2020 because most tools of relevance were predicted to have emerged over this timeframe.

### Step 2: selecting material

Table 2 outlines the complete list of inclusion and exclusion criteria applied to the mapping review. In the process of screening, minor changes were made to definitions and criteria to improve the clarity and consistency for judging material that could be considered ‘tools’ and which were relevant to health systems building blocks and health systems thinking. It was also critical to refine the definition of rural-based tools. It was decided to include results for rural, regional or underserved locations as long as ‘underserviced’ was defined according to geographic locations and it was consistent with the context of rural places. Where there was disagreement, discussion by the research team was undertaken until a clear basis for inclusion or exclusion was determined. This occurred through regular online meetings of the researchers over a 6-month period.

**Table 2:** Inclusion, exclusion criteria

Inclusion criteria	Exclusion criteria
– Last 15 years (2005–2020) – Written in English – Clear methods involving data collection (secondary or primary) (not literature reviews or perspectives), articles where the tool was developed, used, or validated or both – Includes results for rural or underserviced locations, not for a generic geographical context – Studies in regional/non-metropolitan/resource-limited, as long as they provide rural/underserviced definition and the context – Rural or underserved is defined by its distance/geography and limited workforce – Studies that used other terms such as remote, under-serviced, poor-resource and resource-limited – Include differentiated results for rural, whether urban is included in the study or not – Aligns with definition of *framework, tool or resource* (Box 1) – About improving/enhancing health system components using health systems thinking	– Before last 15 years – Non-English – No clear methods involving data collection – No separate results for rural locations – Documents that include descriptive or narrative literature alone, or reports with recommendations that do not fit concept of framework, tool or resource, systems components or system thinking – Conceptual frameworks that are used as reference or as a method to guide the data collection/analysis rather than for planning and decision-making about rural health systems – Government reviews that arrive at principles and actions for specific topics at a point in time and do not specifically guide ongoing dynamic action on “how to” respond to systems issues in rural communities. These tend to focus on particular events, policies, factors or causalities

**Fig. 1:**
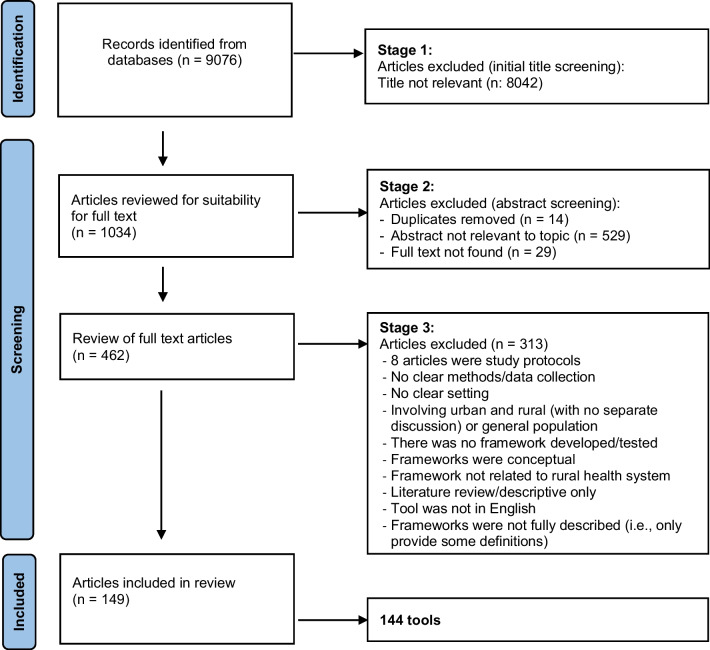
PRISMA flow diagram of reviewed articles

The initial sorting of peer reviewed literature was done by the researchers identifying title and abstracts of relevance. It was necessary to read full text to determine the inclusion of many articles because it was not clear from title/abstract screening that they met the selection criteria. As such, a precautionary approach was used initially in the first level of screening. A second stage of initial screening was applied by the lead author, with regular monitoring by other lead researchers, ensuring consistent application of definitions and criteria. The third stage of screening, led by the main author, involved reviewing the full text articles, aggregating multiple articles on the same tool and removing duplicates. Figure 1 (Prisma diagram) summarises the number of identified and excluded articles at each screening stage.

### Step 3: analysing material

The short-listed literature was mapped out across categories to identify the spread of material and any gaps. The first step was to map the material according to WHO regions and LMIC/HIC and then based on the rural health system building blocks. Next, the data were extracted by year, country of origin, context (stakeholder type, type of service, health worker, rural or remote context, hospital or community), potential users and intended use (proposed function). The scope of extraction was determined based on what information would inform the use of the tools by different audiences. Finally, the methods used to develop the tool were then extracted to allow for a broad quality appraisal of each tool’s development, including whether it had been field tested, validated and/or evaluated.

## Results

The initial literature search generated 9076 articles (Fig. 1). After reviewing the titles, 1034 articles were identified as relevant and abstracts were reviewed. Of these, 14 duplicates were removed, 529 articles were deemed not relevant, and 29 articles could not be found, leaving 462 articles for full text screening. After reading the full texts, 313 articles were excluded because they did not meet the inclusion criteria, leaving 149 included in the review. From 149 articles, there were 144 tools (some tools were reported more than once in different research studies).

### Study settings

Based on the WHO regions, the majority of the tools were identified in the Americas region (*n* = 63, 43%), followed by Africa (*n* = 39, 26%) and Western Pacific (*n* = 34, 23%) (Table 3). Nearly all of the identified tools were from either High- (*n* = 85, 59%) or Middle-income countries (*n* = 43, 29%), with less from Low-income countries (*n* = 17, 12%) (Table 3). Tools from Americas and Western Pacific regions were predominantly from HIC (88% and 89%, respectively). The majority of the studies used the term ‘rural’ (120 tools, 83%) to describe their study location and the remainder used substitute terms like ‘remote’, ‘regional’ or ‘underserviced’.

**Table 3:** Study settings of tools based on the WHO regions and World Bank category

WHO regions	Number of tools	%
Americas	63	43
Africa	39	26
Western Pacific	34	23
South-East Asia	9	6
Eastern Mediterranean	1	1
Europe	1	1
Sub total	147^a^	100

Category of country		%
High income countries (HIC)	85	59
Middle income countries (MIC)	43	29
Low income countries (LIC)	17	12
Sub total	145^b^	100
Total	144	100

- Nursing Community Apgar Questionnaire (NCAQ) [11, 12]: America and Western Pacific.

- Virtual Community of Practice (VCoP) [13–15]: Africa and Western Pacific.

- Community-Centred Practice Framework (CCPF) [16]: America and Western Pacific.

^a^Three tools were identified in two different regions: - NCAQ [11, 12], - VCoP [13–15], - CCPF [16]

^b^One tool was developed in HIC and LMIC (VCoP): [13–15].

## Health system blocks

Most (*n* = 106, 74%) tools concerned one health system block only, whereas 38 (26%) related to more than one health system block (‘combined’). Most single block tools related to the service building block (approaches to continuity of care, service integration, models of care) (*n* = 57, 39%), or workforce (training, quality improvement for performance, recruitment and retention) (*n* = 33, 23%) (Table 4). When combined tools were covered, this increased to 90 (63%) and 40 tools (28%), respectively for these health system blocks. There were no single block tools concerning infrastructure or financing and few related to this when combined tools were considered. The trends are similar both in HICs and LMICs, in terms of the proportion of tools developed in each health system block (Additional files 1, 2).

**Table 4:** Numbers of tools in each health system compared in HIC and LMIC

Health system block	Number of tools in HIC	Number of tools in LMIC	Total	%
Single				
Services	35	22	57	39
Workforce	19^a^	15^a^	34^a^	23
Information	5	3	8	5
Leadership and governance	3	5	8	5
Infrastructure	0	0	0	0
Financing	0	0	0	0
Combined				
Service, information	9	6	15	10
Service, leadership and governance	7	3	10	7
Service, workforce	3	1	4	3
Leadership and governance, workforce	1	2	3	2
Information, financing	1	0	1	1
Information, service, leadership and governance	0	1	1	1
Infrastructure, financing, leadership and governance	0	1	1	1
Service, workforce, information, leadership and governance	1	0	1	1
All health system blocks	1	1	2	1
Total	85	60	145^a^	100

^a^One tool was developed in HIC and LMIC (VCoP): [13–15]

### Services

Tools related to strengthening rural health services (*n* = 90, 62%) were mostly designed for a specific disease (*n* = 38, 43%), the majority being non-communicable/degenerative diseases such as heart failure [17–19], hypertension [20, 21], stroke [22], cancer [23–25], kidney disease [26], diabetes [27–30], and mental health issues [31–38]. Few tools related to communicable/infectious diseases, such as HIV/AIDS [39–43] and malaria [44, 45]. Other tools were targeted to varied life-stages; maternal and neonatal [46–53], children [54–56], youth [57], adults and elder people health [58–63]. Few tools concerned food and nutrition [64–66], gender equality [67], oral health [68], sexual health [69] and palliative care [70–72].

### Workforce

Of the 40 (28%) tools concerning workforce, most commonly these were related to doctors (*n* = 12, 30%), followed by nurses (*n* = 10, 25%) (Table 5). The rest of the tools were developed for a variety of health professionals, medical students, community health workers and for community members (layperson).

**Table 5:** Tools related to workforce

Health professional	Number of tools	%
Doctor	12	30
Nurse	10	25
Rural healthcare workers	3	7
Non- physician clinicians (NPCs)	3	7
Allied health professionals	2	5
Surgeons	2	5
Paediatricians	1	2
Counsellors	1	2
Health manager	1	2
Medical students	1	2
Nutrition professionals	1	2
Paramedic	1	2
Social worker	1	2
Rural private therapists	1	2
Community healthcare worker	1	2
Acute Care Providers (community—layperson)	1	2
Total	42^a^	100

^a^There are two tools that were developed for two health professionals [73, 74]

The majority of tools in the workforce block were developed for promoting health professionals’ education and increasing their competencies [52, 75–95]. Some tools related to delivering training, such as virtual Information Communication Technology (ICT) training for doctors [75], training on point-of-care ultrasound (POCUS) for non-physician health providers [76], Physician Management and Leadership Program (PMLP) [77] and the ALL BABIES COUNT (ABC) initiative [52]. Other tools provided curriculum and competency frameworks for rural healthcare workers [78–80]. One tool also related to culturally relevant online learning for rural workers [87]. Other tools aimed to inform training of community members to provide basic health care [96] and to help community healthcare workers to communicate with maternal care clients during home visits [88].

Others related to increasing the capacity of rural educational placements, including a tool informing a collaborative model of clinical education [92], developed rural hospital and education providers. TeleOSCE was developed for assessing the clinical skills of medical students in rural rotation [93], and another framework was applied for preceptor evaluation to evaluate student performance in rural setting [94]. One tool further related to increasing competencies of social worker students on Rural Child Welfare [95]. Other tools included those targeting supervision capacity for allied health professionals [97], non-physician clinicians and medical doctors [73], and nurses [98].

Professional support tools were also noted. These included those for doctors through Virtual Communities of Practice (VCoP), an online community to support knowledge sharing in the general practice training community [14, 15]. There is also a tool for a multi-faceted view of actions needed to support auxiliary nurses’ performance [99], support paramedic in the community (COPE—Community support, Organisational support, Professional Support, Education and Training multidisciplinary practice) [100], support for rural private therapists [101], and support staff occupational safety and wellness [102].

In terms of attracting and retaining health workers one framework aimed to understand personal factors that contribute to the potential for living in rural areas [103], another preliminary framework informed action related to physician retention [104]. There was a tool about a mentorship program to support nurses transition to rural communities [105], a tool related to choice experiment (CE) attributes [74], and a framework for locally relevant training to increase retention [91]. The nursing community APGAR questionnaire was developed [11] and tested [12] to measure factors that affect rural nurse recruitment and retention.

### Information

There were 26 tools that aimed to improve rural health system information (electronic record keeping, data systems and information gathering), most of which involved health records enhancement. The development of information systems in rural areas included a tool concerning the use of cloud-based health information centres or databases that provided access to healthcare services remotely [72, 106, 107]. One tool was developed specifically to help improve medication administration, in order to reduce errors [108, 109]. Clinical decision support tools were also identified which aimed to guide action to improve patient outcomes in rural healthcare [51, 63, 110]. Two tools related to informing the use of mobile/phone applications; one tool related to using a WhatsApp group to support communication and distributed information in rural health work [111] and the other one was The SPIRIT app mobile system, designed to support the effective delivery of Collaborative Care for people with Posttraumatic Stress Disorder and bipolar disorder [36].

### Leadership and governance

There were 24 tools designed for leadership and governance (decentralisation, community participation, workforce licensure, accreditation and registration) of rural health system development. In rural settings, community was considered to be an essential source of support to improve health system, that the identified tools had a variety of aims including increasing community participation [112], community-initiated health service partnership [113], community-based referral system [114], community mobilisation [53, 115], measuring community participation [116], and a sustainable community transport system [117]. Some tools were developed to strengthen partnerships/collaborations building between academic institutions and the community through Strong Rural Communities Initiative (SRCI) [118], between international and three community partners in Guatemala [119], and as part of a multi-institution partnership model [23].

### Infrastructure

There was only one tool identified in this review that related to improved infrastructure in rural health systems (supply of materials and equipment, system architecture for integrated delivery of interventions). This tool was developed to create a community driven sustainable transportation system to support referral and health access for maternal health care services in rural Ghana [117]. This tool not only aimed to inform improvements of infrastructure (transportation), but also to inform increased community participation (leadership and governance block). This tool conceptualized two components of transportation systems, which were the source of funding for transport, and the management of transport operation. Hence, this tool also related to financing of the health system.

### Financing

There were two tools that related to financing block (remuneration, insurance, incentives) of rural health system. The first tool was called the Patient Classification System (PCS), which helped nurse managers to monitor cost and improve patient care [120]. This tool helped nurses to indicate the most suitable patient care resources in rural settings. The second tool related to financing a sustainable community transport system for rural health access, as described above [117].

## Quality of the tools

**Fig. 2:**
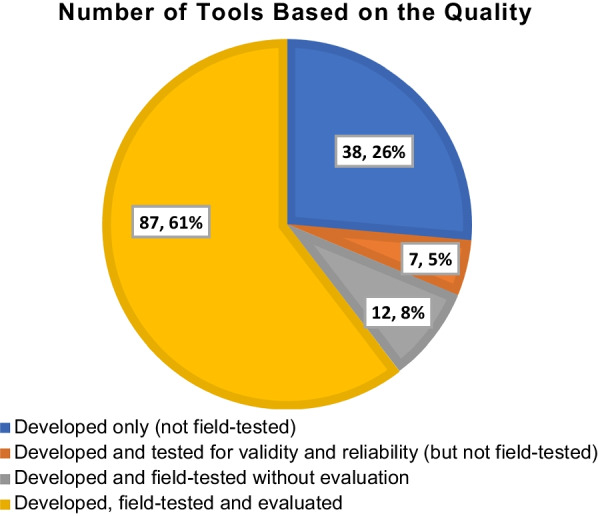
Tools quality

The majority of the tools (n = 87, 61%) included in this review had been field-tested and evaluated, whilst many (n = 38, 26%) had been developed without any identified field-testing (Fig. 2). Few tools incorporated specific validity and reliability testing (5%).

## Discussion

This mapping review identified a range of useful tools that may be relevant to support action on rural health system strengthening across the world. Increasing awareness and access to these tools in various countries and rural and remote contexts, could significantly enhance coordinated planning for adaptable rural health system developments to meet current and emerging needs. The findings also showed where there are gaps in the numbers of tools developed across different regions in HICs and LMICs as well as gaps in the number and variety of tools developed for each block of the health systems. This is important for being able to showcase gaps that need to be addressed to support rural health system development across the spectrum of health system building blocks for more comprehensive effects. Further, many tools are specific to particular service types, or types of health workers which may limit their generalisability. This is particularly true where countries use different cadres and types of health workers, health care teams and volunteers in their rural health systems. It would be helpful if more tools encompassed a wider range of health workers or a team-based approach in the development phase, rather than relying on adapting a tool that was developed for one type of health worker to meet the needs of another after the production phase.

This review showed that the majority of the tools were developed, tested and applied in HIC. This may not be surprising, because funding for tool development research might be more available in HICs. In addition to funding, HICs have more resources, access to technical equipment, technology-literate staff and better funded infrastructure. The gaps in the availability of tools in LMIC suggests that collaboration in research is important and the WHO and other agencies should encourage more global networks to work together when developing tools for rural health system strengthening. This review identified many tools related to technology which aimed to improve rural service, workforce and information blocks. These included telehealth [22, 25, 43, 46, 89, 93], development of health-related website [24, 28, 63, 121] and developing electronic health records [17, 107, 108]. There are some factors that might influence the readiness of rural health systems to implement and apply telehealth successfully, including managing resistance to change, and the efficiency of the technology introduced [122]. While technology has been a substantial focus of improving rural health systems, there should be consideration as to effective it can be where there is limited available of local health workers, resources or skills, to wrap around such solutions. Gaps of the number of tools in LMICs and HICs was also seen in workforce block, with more tools identified that were developed and applied in this area within HIC compared to LMIC (25 vs. 16). It is critical to address this gap given the prominence of workforce shortages globally, but being most extreme in the LMIC context. It is possible that LMICs have access to tools that have not been published in peer-reviewed journals due to time and resources pressures, and one option is to increase partnerships between global partners to assist with publication processes.

A primary finding identified in this review is that there were imbalances in the number of tools that had been developed in each health system block. The majority of tools were developed for only one of the service and workforce blocks, whilst notably there were few tools relating to either infrastructure or financing. This suggests there is likely to be uneven approaches to rural health system strengthening and if not comprehensive, then health system strengthening will not be effective. Although the shortage of healthcare providers is the most common challenges in rural areas, the whole rural health system relies on all of the system blocks supporting each other and reinforcing resilience and adaptability. Thus, it is important to fill the gaps in some of the health system building blocks and make the suite of tools and resources more readily available to users, perhaps by creating a catalogue. This review suggests that there is an urgent need to also increase the number of studies that develop and validate tools in the other health system blocks, particularly those that include information, leadership and governance, financing and infrastructure.

In addition, this review also found that the majority of the tools have been implemented and evaluated at the time of the review was conducted, with around 26% of the developed tools having no evidence of further testing and evaluation. This suggests that many more require ongoing validation before they can be applied. Further, it is important that existing tools undergo field-testing in a range of contexts, not limited to one country, because of the variability of rural settings and conditions which could impact suitability of any one tool for rural health system strengthening.

## Limitations

By necessity, this review was constrained to a fixed time period but it is acknowledged that new tools are regularly emerging or replacing older ones. Furthermore, it is acknowledged that many relevant tools may exist but are not published in peer-review journals, thus out of scope for this review. While focusing on peer reviewed evidence aimed to obtain high quality articles for the review, the exclusion of grey literature might result in a narrower scope of materials, and thereby a less comprehensive view of the available evidence [123]. There was a possibility that by excluding grey literature, the researchers might have missed relevant material. No data were collected of the popularity or breadth of application of the various tools; thus, no judgement was made of their usefulness or impact. Whilst the quality of each tool’s development is included, this was not linked to subsequent applications or validation of the tool in other studies. Great care was given to the clarity of definitions relating to both what constitutes a tool (frameworks, tools and resources) and how such a tool relates to the health system; however, it is acknowledged this review may have some gaps relating to subjective decisions being applied.

## Conclusions

This mapping review of published tools that build rural health systems provides important evidence on the breadth of tools available globally, the regional context of their development and the gaps identified across the WHO health system building blocks. The findings suggest that as most tools were developed and applied in HIC, it is important to consider factors that influence (both positively and negatively) their utility when applying the tools in LMIC settings. Most tools identified in this review were developed for services and workforce, with tools for information and leadership and governance development also well represented. Very few were identified for developing infrastructure or financing. This suggests that there is strong need to develop tools related to these underrepresented system blocks, given their potential to impact on the other system blocks and overall strengthening of rural health systems. With rural communities being different in characteristics, applying one tool that has been developed, tested and applied in other rural settings will likely require consideration of the local context. Hence, prior to application, tools may need re-testing and re-development in accordance with local needs.

### Supplementary Information

**Additional file 1: Appendix 1: **Articles used in the review (n = 149).

**Additional file 2:** Summaries of articles reviewed in each of the health system building block.

## Data Availability

Not applicable.

## Abbreviations

HIC: High income countries
LMIC: Low- and middle-income countries
