# Supplementary material for: Tools, frameworks and resources to guide global action on strengthening rural health systems: a mapping review
Source: Health Res Policy Syst. 2023 Dec 4;21:129. doi: 10.1186/s12961-023-01078-3 (PMC10694960; doi:10.1186/s12961-023-01078-3)
Supplement: Supplementary file 1 — Additional file 1: Appendix 1: Articles used in the review (n = 149). [file 12961_2023_1078_MOESM1_ESM.docx]

**Appendix I: Articles used in the review (n= 149)**

1. Abbey M, Bartholomew LK, Chinbuah MA, Gyapong M, Gyapong JO, van den Borne B. Development of a theory and evidence-based program to promote community treatment of fevers in children under five in a rural district in Southern Ghana: An intervention mapping approach. BMC Public Health. 2017;17(1):1-11.

2. Adcock AK, Choi J, Alvi M, Murray A, Seachrist E, Smith M, et al. Expanding Acute Stroke Care in Rural America: A Model for Statewide Success. Telemed J E Health. 2019;09:09.

3. Agarwal M, Bourgeois J, Sodhi S, Matengeni A, Bezanson K, van Schoor V, et al. Updating a patient-level ART database covering remote health facilities in Zomba district, Malawi: lessons learned. Public health action. 2013;3(2):175-9.

4. Ahmed SM, Size T, Crouse B, Patterson L, Gass E, Karon SL, et al. Strong Rural Communities Initiative (SRCI) program: challenges in promoting healthier lifestyles. Wmj. 2011;110(3):119-26.

5. Aleem S, Torrey WC, Duncan MS, Hort SJ, Mecchella JN. Depression screening optimization in an academic rural setting. Int J Health Care Qual Assur. 2015;28(7):709-25.

6. Ansinelli DJ. The Effectiveness of a Structured Telephone Support Program for Rural Patients with Heart Failure: West Virginia University; 2013.

7. Atuoye KN, Dixon J, Rishworth A, Galaa SZ, Boamah SA, Luginaah I. Can she make it? Transportation barriers to accessing maternal and child health care services in rural Ghana. BMC Health Serv Res. 2015;15:333.

8. Baatiema L, Skovdal M, Rifkin S, Campbell C. Assessing participation in a community-based health planning and services programme in Ghana. BMC Health Serv Res. 2013;13:233.

9. Barnett S, Jones SC, Bennett S, Iverson D, Bonney A. Usefulness of a virtual community of practice and Web 2.0 tools for general practice training: experiences and expectations of general practitioner registrars and supervisors. Aust J Prim Health. 2013;19(4):292-6.

10. Barnett S, Jones SC, Caton T, Iverson D, Bennett S, Robinson L. Implementing a virtual community of practice for family physician training: a mixed-methods case study. J Med Internet Res. 2014;16(3):e83.

11. Barnett T, Cross M, Shahwan-Akl L, Jacob E. The evaluation of a successful collaborative education model to expand student clinical placements. Nurse Educ Pract. 2010;10(1):17-21.

12. Baron KP. Incorporating Personal Health Records into the Disease Management of Rural Heart Failure Patients 2012.

13. Bauer AM, Hodsdon S, Bechtel JM, Fortney JC. Applying the Principles for Digital Development: Case Study of a Smartphone App to Support Collaborative Care for Rural Patients With Posttraumatic Stress Disorder or Bipolar Disorder. J Med Internet Res. 2018;20(6):e10048.

14. Beck K, Mukantaganda A, Bayitondere S, Ndikuriyo R, Dushimirimana A, Bihibindi V, et al. Experience: developing an inpatient malnutrition checklist for children 6 to 59 months to improve WHO protocol adherence and facilitate quality improvement in a low-resource setting. Glob Health Action. 2018;11(1):1503785.

15. Bedard TE, Nadin S, Zufelt C, Cheng C. Implementation and evaluation of a quality improvement project: carepaths for Early Psychosis Intervention Programmes in Northeastern Ontario. Early Interv Psychiatry. 2016;10(6):547-53.

16. Bess KD, Frerichs L, Young T, Corbie-Smith G, Dave G, Davis K, et al. Adaptation of an Evidence-Based Cardiovascular Health Intervention for Rural African Americans in the Southeast. Prog. 2019;13(4):385-96.

17. Birks L, Powell C, Hatfield J. Adapting the capacities and vulnerabilities approach: a gender analysis tool. Health Promot Internation. 2017;32(6):930-41.

18. Biziyaremye F, Nahimana E, Mutaganzwa C, Tugizimana D, Werdenberg J, Magge H, et al. Successful implementation of a combined learning collaborative and mentoring intervention to improve neonatal quality of care in rural Rwanda. BMC Health Serv Res. 2018;18(1):941-.

19. Blank E, Tuikong N, Misoi L, Kamano J, Hutchinson C, Kimaiyo S, et al. Usability of Implementing a Tablet-Based Decision Support and Integrated Record- Keeping (DESIRE) Tool in the Nurse Management of Hypertension in Rural Kenya. Stud Health Technol Inform. 2013;192:1002-.

20. Bonsignore L, Bloom N, Steinhauser K, Nichols R, Allen T, Twaddle M, et al. Evaluating the Feasibility and Acceptability of a Telehealth Program in a Rural Palliative Care Population: TapCloud for Palliative Care. J Pain Symptom Manage. 2018;56(1):7-14.

21. Bontempo T, Westmacott L, Paterson J, Paterson M. The development of a resource guide on post traumatic stress disorder for rural health care workers. Asia Pacific Disability Rehabilitation Journal. 2008;19(2):34-49.

22. Brimblecombe J, van den Boogaard C, Wood B, Liberato SC, Brown J, Barnes A, et al. Development of the good food planning tool: A food system approach to food security in indigenous Australian remote communities. Health Place. 2015;34:54-62.

23. Brokel JM, Schwichtenberg TJ, Wakefield DS, Ward MM, Shaw MG, Kramer JM. Evaluating clinical decision support rules as an intervention in clinical workflows with technology. Comput Inform Nurs. 2011;29(1):36-42.

24. Brooks GL. Improving the management of patients with type-2 diabetes in a rural clinic: University of Arizona; 2011.

25. Brown JB, Morrison T, Bryant M, Kassell L, Nestel D. A framework for developing rural academic general practices: a qualitative case study in rural Victoria. Rural Remote Health. 2015;15(2):3072.

26. Butterfield P, Postma J, team Er. The TERRA framework: conceptualizing rural environmental health inequities through an environmental justice lens. ANS Adv Nurs Sci. 2009;32(2):107-17.

27. Calano BJD, Cacal MJB, Cal CB, Calletor KP, Guce FICC, Bongar MVV, et al. Effectiveness of a community‐based health programme on the blood pressure control, adherence and knowledge of adults with hypertension: A PRECEDE‐PROCEED model approach. Journal of Clinical Nursing (John Wiley & Sons, Inc). 2019;28(9/10):1879-88.

28. Cameron PJ, Este DC, Worthington CA. Professional, personal and community: 3 domains of physician retention in rural communities. Can J Rural Med. 2012;17(2):47-55.

29. Campbell J, Aturinda I, Mwesigwa E, Burns B, Santorino D, Haberer J, et al. The Technology Acceptance Model for Resource-Limited Settings (TAM-RLS): A Novel Framework for Mobile Health Interventions Targeted to Low-Literacy End-Users in Resource-Limited Settings. Aids Behav. 2017;21(11):3129-40.

30. Carlisle K, Farmer J, Taylor J, Larkins S, Evans R. Evaluating community participation: A comparison of participatory approaches in the planning and implementation of new primary health-care services in northern Australia. Int J Health Plan Manag. 2018;33(3):704-22.

31. Chen N, Hsieh HP, Tsai RK, Sheu MM. Eye care services for the populations of remote districts in eastern Taiwan: a practical framework using a Mobile Vision Van Unit. Rural Remote Health. 2015;15(4):3442.

32. Cherrington A, Martin MY, Hayes M, Halanych JH, Wright MA, Appel SJ, et al. Intervention mapping as a guide for the development of a diabetes peer support intervention in rural Alabama. Prev Chronic Dis. 2012;9:E36.

33. Citrin D, Thapa P, Nirola I, Pandey S, Kunwar LB, Tenpa J, et al. Developing and deploying a community healthcare worker-driven, digitally-enabled integrated care system for municipalities in rural Nepal. HealthCare. 2018;6(3):197-204.

34. Clancy A. Practice model for a dementia outreach service in rural Australia. Aust J Rural Health. 2015;23(2):87-94.

35. Coleman A. A Virtual Community of Practice Framework to Support Doctors' Practices in National Health Insurance (NHI) in South Africa. Stud Ethno-Med. 2012;6(3):155-60.

36. Coleman A. Using a Virtual ICT Training Framework to Support Doctors in Rural Hospitals in South Africa. Stud Ethno-Med. 2013;7(3):137-41.

37. Coleman A, Akinsola OS. Effective Blood Distribution in Rural Hospitals through ICT Service Oriented Architecture (SOA) Framework: A Case Study in Rural Hospitals in South Africa. Stud Ethno-Med. 2012;6(3):141-7.

38. Connelly J, Kirk A, Masthoff J, MacRury S. A Website to Promote Physical Activity in People With Type 2 Diabetes Living in Remote or Rural Locations: Feasibility Pilot Randomized Controlled Trial. JMIR Diabetes. 2017;2(2):e26.

39. Conway P, Favet H, Hall L, Uhrich J, Palcher J, Olimb S, et al. Rural Health Networks and Care Coordination: Health Care Innovation in Frontier Communities to Improve Patient Outcomes and Reduce Health Care Costs. J Health Care Poor Underserved. 2016;27(4):91-115.

40. Cueva K, Cueva M, Revels L, Lanier AP, Dignan M, Viswanath K, et al. A Framework for Culturally Relevant Online Learning: Lessons from Alaska's Tribal Health Workers. J Cancer Educ. 2019;34(4):647-53.

41. Daivadanam M, Ravindran TKS, Thankappan KR, Sarma PS, Wahlstrom R. Development of a Tool to Stage Households' Readiness to Change Dietary Behaviours in Kerala, India. PLoS ONE. 2016;11(11):13.

42. de la Torre A. Benevolent Paradox: Integrating Community-Based Empowerment and Transdisciplinary Research Approaches into Traditional Frameworks to Increase Funding and Long-Term Sustainability of Chicano-Community Research Programs. SAGE Publications, 2455 Teller Road, Thousand Oaks, CA 91320; 2014 Apr 2014.

43. Dent E, Hoon E, Kitson A, Karnon J, Newbury J, Harvey G, et al. Translating a health service intervention into a rural setting: lessons learned. BMC Health Serv Res. 2016;16:62.

44. Dew A, Barton R, Ragen J, Bulkeley K, Iljadica A, Chedid R, et al. The development of a framework for high-quality, sustainable and accessible rural private therapy under the Australian National Disability Insurance Scheme. Disabil Rehabil. 2016;38(25):2491-503.

45. Ding Y, Sauerborn R, Xu B, Shaofa N, Yan W, Diwan VK, et al. A cost-effectiveness analysis of three components of a syndromic surveillance system for the early warning of epidemics in rural China. BMC Public Health. 2015;15:1127.

46. Downing J. The conception of the Nankya model of palliative care development in Africa. Int J Palliat Nurs. 2008;14(9):459-64.

47. Ducat W, Martin P, Kumar S, Burge V, Abernathy L. Oceans apart, yet connected: Findings from a qualitative study on professional supervision in rural and remote allied health services. Aust J Rural Health. 2016;24(1):29-35.

48. English M, Nzinga J, Mbindyo P, Ayieko P, Irimu G, Mbaabu L. Explaining the effects of a multifaceted intervention to improve inpatient care in rural Kenyan hospitals - interpretation based on retrospective examination of data from participant observation, quantitative and qualitative studies. Implement Sci. 2011;6:12.

49. Ens CD, Hanlon-Dearman A, Millar MC, Longstaffe S. Using telehealth for assessment of fetal alcohol spectrum disorder: the experience of two Canadian rural and remote communities. Telemed J E Health. 2010;16(8):872-7.

50. Fares J, Chung KSK, Passey M, Longman J, Valentijn PP. Exploring the psychometric properties of the Rainbow Model of Integrated Care measurement tool for care providers in Australia. BMJ Open. 2019;9(12):8.

51. Fekadu A, Hanlon C, Medhin G, Alem A, Selamu M, Giorgis TW, et al. Development of a scalable mental healthcare plan for a rural district in Ethiopia. Br J Psychiatry. 2016;208 Suppl 56:s4-12.

52. Fekadu A, Medhin G, Lund C, DeSilva M, Selamu M, Alem A, et al. The psychosis treatment gap and its consequences in rural Ethiopia. BMC Psychiatry. 2019;19(1):325.

53. Feng S, Shi L, Zeng J, Chen W, Ling L. Comparison of Primary Care Experiences in Village Clinics with Different Ownership Models in Guangdong Province, China. PLoS ONE. 2017;12(1):e0169241.

54. Fennell KM, Turnbull DA, Bidargaddi N, McWha JL, Davies M, Olver I. The consumer-driven development and acceptability testing of a website designed to connect rural cancer patients and their families, carers and health professionals with appropriate information and psychosocial support. Eur J Cancer Care (Engl). 2017;26(5):n/a-N.PAG.

55. Fialkowski M, DeBaryshe B, Bersamin A, Nigg C, Leon Guerrero R, Rojas G, et al. A Community Engagement Process Identifies Environmental Priorities to Prevent Early Childhood Obesity: The Children's Healthy Living (CHL) Program for Remote Underserved Populations in the US Affiliated Pacific Islands, Hawaii and Alaska. Matern Child Health J. 2014;18(10):2261-74.

56. Flood D, Douglas K, Goldberg V, Martinez B, Garcia P, Arbour M, et al. A quality improvement project using statistical process control methods for type 2 diabetes control in a resource-limited setting. Int J Qual Health Care. 2017;29(4):593-601.

57. Fortney JC, Pyne JM, Edlund MJ, Robinson DE, Mittal D, Henderson KL. Design and implementation of the Telemedicine-Enhanced Antidepressant Management study. Gen Hosp Psychiatry. 2006;28(1):18-26.

58. Foster AA, Makukula MK, Moore C, Chizuni NL, Goma F, Myles A, et al. Strengthening and Institutionalizing the Leadership and Management Role of Frontline Nurses to Advance Universal Health Coverage in Zambia. Glob. 2018;6(4):736-46.

59. Fox A, Beyers J. Planning a graduate programme in public health nutrition for experienced nutrition professionals. Public Health Nutr. 2011;14(8):1479-88.

60. Fragar L, Kelly B, Peters M, Henderson A, Tonna A. Partnerships to promote mental health of NSW farmers: The new south wales farmers blueprint for mental health. Aust J Rural Health. 2008;16(3):170-5.

61. Fujita M, Poudel KC, Do TN, Bui DD, Nguyen VK, Green K, et al. A new analytical framework of 'continuum of prevention and care' to maximize HIV case detection and retention in care in Vietnam. BMC Health Serv Res. 2012;12:483.

62. Gajewski J, Monzer N, Pittalis C, Bijlmakers L, Cheelo M, Kachimba J, et al. Supervision as a tool for building surgical capacity of district hospitals: the case of Zambia. Hum Resour Health. 2020;18(1):25.

63. Gerolamo A, Kim J, Brown J, Schuster J, Kogan J, Gerolamo AM, et al. Implementation of a Reverse Colocation Model: Lessons from Two Community Behavioral Health Agencies in Rural Pennsylvania. J Behav Health Serv Res. 2016;43(3):443-58.

64. Give C, Ndima S, Steege R, Ormel H, McCollum R, Theobald S, et al. Strengthening referral systems in community health programs: a qualitative study in two rural districts of Maputo Province, Mozambique. BMC Health Serv Res. 2019;19(1):N.PAG-N.PAG.

65. Goodman M, Almon L, Bayakly R, Butler S, Crosby C, DiIorio C, et al. Cancer outcomes research in a rural area: a multi-institution partnership model. J Community Health. 2009;34(1):23-32.

66. Gouveia EA, Braga TD, Heraclio SA, Pessoa BH. Validating competencies for an undergraduate training program in rural medicine using the Delphi technique. Rural Remote Health. 2016;16(4):3851.

67. Grande KM, Stanley M, Redo C, Wergin A, Guilfoyle S, Gasiorowicz M. Social Network Diagramming as an Applied Tool for Public Health: Lessons Learned From an HCV Cluster. Am J Public Health. 2015;105(8):1611-6.

68. Harper K, McCully C. Acuity systems dialogue and patient classification system essentials. Nurs Adm Q. 2007;31(4):284-99.

69. Hearns G, Klein MC, Trousdale W, Ulrich C, Butcher D, Miewald C, et al. Development of a support tool for complex decision-making in the provision of rural maternity care. Healthc Policy. 2010;5(3):82-96.

70. Hernandez AR, Hurtig AK, Dahlblom K, San Sebastian M. Integrating views on support for mid-level health worker performance: a concept mapping study with regional health system actors in rural Guatemala. Intern. 2015;14:91.

71. Heslop CW, Burns S, Lobo R, McConigley R. Developing a framework for community-based sexual health interventions for youth in the rural setting: protocol for a participatory action research study. BMJ Open. 2017;7(5):e013368.

72. Hetzel MW, Iteba N, Makemba A, Mshana C, Lengeler C, Obrist B, et al. Understanding and improving access to prompt and effective malaria treatment and care in rural Tanzania: the ACCESS Programme. Malar J. 2007;6:83.

73. Hinton R, Kavanagh DJ, Barclay L, Chenhall R, Nagel T. Developing a best practice pathway to support improvements in Indigenous Australians' mental health and well-being: a qualitative study. BMJ Open. 2015;5(8):e007938.

74. Hoffman AS, Bateman DR, Ganoe C, Punjasthitkul S, Das AK, Hoffman DB, et al. Development and Field Testing of a Long-Term Care Decision Aid Website for Older Adults: Engaging Patients and Caregivers in User-Centered Design. Gerontologist. 2019;27:27.

75. Hogan RM. Re-engineered discharge planning in a rural Mississippi hospital to reduce 30 day readmission rates among heart failure patients: University of Southern Mississippi; 2014.

76. Honda A, Krucien N, Ryan M, Diouf ISN, Salla M, Nagai M, et al. For more than money: willingness of health professionals to stay in remote Senegal. Human Resources for Health. 2019;17(1):N.PAG-N.PAG.

77. Hounton S, Byass P, Brahima B. Towards reduction of maternal and perinatal mortality in rural Burkina Faso: communities are not empty vessels. Global Health Action. 2009;2(1):1-9.

78. Huang Y-H. Evolution of Medication Administration Workflow in Implementing Electronic Health Record System 2013.

79. Huang Y-H, Gramopadhye AK. Systematic engineering tools for describing and improving medication administration processes at rural healthcare facilities. Applied Ergonomics. 2014;45(6):1712-24.

80. Hyett N, Kenny A, Dickson-Swift V. Re-imagining occupational therapy clients as communities: Presenting the community-centred practice framework. Scand J Occup Ther. 2019;26(4):246-60.

81. Idoga PE, Toycan M, Nadiri H, Celebi E. Factors Affecting the Successful Adoption of e-Health Cloud Based Health System From Healthcare Consumers' Perspective. IEEE Access. 2018;6:71216-28.

82. Im DD, Palazuelos L, Xu L, Molina RL, Palazuelos D, Sullivan MM. A Community-Based Approach to Cervical Cancer Prevention: Lessons Learned in Rural Guatemala. Prog. 2018;12(1):45-54.

83. Isler J, Sawadogo NH, Harling G, Barnighausen T, Adam M, Kagone M, et al. Iterative Adaptation of a Maternal Nutrition Videos mHealth Intervention Across Countries Using Human-Centered Design: Qualitative Study. JMIR Mhealth Uhealth. 2019;7(11):e13604.

84. Joarder T, Mahmud I, Sarker M, George A, Rao KD. Development and validation of a structured observation scale to measure responsiveness of physicians in rural Bangladesh. BMC Health Serv Res. 2017;17(1):753.

85. Kelley ML. Developing rural communities' capacity for palliative care: a conceptual model. J Palliat Care. 2007;23(3):143-53.

86. Kelley ML, Williams A, DeMiglio L, Mettam H. Developing rural palliative care: validating a conceptual model. Rural Remote Health. 2011;11(2):1717.

87. Khan A, Sebok-Syer SS, Linstadt H, Storm M, Modan N, Bosco MK, et al. An Electronic-Based Curriculum to Train Acute Care Providers in Rural Haiti and India. J Grad Med Educ. 2019;11(4 Suppl):152-7.

88. Kijima T, Akai K, Matsushita A, Hamano T, Onoda K, Yano S, et al. Development of the Japanese version of the general practice assessment questionnaire: measurement of patient experience and testing of data quality. BMC Fam Pract. 2018;19(1):N.PAG-N.PAG.

89. Kim G, Griffin S, Nadem H, Aria J, Lawry L. Evaluation of an interactive electronic health education tool in rural Afghanistan. Prehospital Disaster Med. 2008;23(3):218-26.

90. Knight A, Havard A, Shakeshaft A, Maple M, Snijder M, Shakeshaft B. The Feasibility of Embedding Data Collection into the Routine Service Delivery of a Multi-Component Program for High-Risk Young People. Int J Environ Res Public Health. 2017;14(2):20.

91. Kram SL, DiBartolo MC, Hinderer K, Jones RA. Implementation of the ABCDE Bundle to Improve Patient Outcomes in the Intensive Care Unit in a Rural Community Hospital. Dimensions of Critical Care Nursing. 2015;34(5):250-8.

92. Krause DD. State Health Mapper: An Interactive, Web-Based Tool for Physician Workforce Planning, Recruitment, and Health Services Research. South Med J. 2015;108(11):650-6.

93. Krishnan A, Nongkynrih B, Yadav K, Singh S, Gupta V. Evaluation of computerized health management information system for primary health care in rural India. BMC Health Serv Res. 2010;10:310.

94. Kuziemsky C, Jewers H, Appleby B, Foshay N, Maccaull W, Miller K, et al. Information technology and hospice palliative care: social, cultural, ethical and technical implications in a rural setting. Inform Health Soc Care. 2012;37(1):37-50.

95. Laurenzi CA, Gordon S, Skeen S, Coetzee BJ, Bishop J, Chademana E, et al. The home visit communication skills inventory: Piloting a tool to measure community health worker fidelity to training in rural South Africa. Res Nurs Health. 2020;43(1):122-33.

96. Lin IB, Beattie N, Spitz S, Ellis A. Developing competencies for remote and rural senior allied health professionals in Western Australia. Rural Remote Health. 2009;9(2):1115.

97. Lippman SA, Neilands TB, Leslie HH, Maman S, MacPhail C, Twine R, et al. Development, validation, and performance of a scale to measure community mobilization. Soc Sci Med. 2016;157:127-37.

98. Maddalena V, Fleet L. Developing a Physician Management & Leadership Program (PMLP) in Newfoundland and Labrador. Leadersh Health Serv (Bradf Engl). 2015;28(1):35-42.

99. Magge H, Anatole M, Cyamatare FR, Mezzacappa C, Nkikabahizi F, Niyonzima S, et al. Mentoring and quality improvement strengthen integrated management of childhood illness implementation in rural Rwanda. Arch Dis Child. 2015;100(6):565-70.

100. Mannik J, Figol A, Churchill V, Aw J, Francis S, Karino E, et al. Community-based screening for cardiovascular risk using a novel mHealth tool in rural Kenya. J Innov Health Inform. 2018;25(3):176-82.

101. Matin SB, Wallingford A, Xu S, Ng N, Ho A, Vanosdoll M, et al. Feasibility of a Mobile Health Tool for Mothers to Identify Neonatal Illness in Rural Uganda: Acceptability Study. JMIR Mhealth Uhealth. 2020;8(2):e16426.

102. McConnel FB, Pashen D, McLean R. The ARTS of risk management in rural and remote medicine...assessment, resources, transport and support. Canadian Journal of Rural Medicine (Joule Inc). 2007;12(4):231-8.

103. McCreight MS, Gilmartin HM, Leonard CA, Mayberry AL, Kelley LR, Lippmann BK, et al. Practical Use of Process Mapping to Guide Implementation of a Care Coordination Program for Rural Veterans. J Gen Intern Med. 2019;34:67-74.

104. McDonald EL, Bailie R, Michel T. Development and trialling of a tool to support a systems approach to improve social determinants of health in rural and remote Australian communities: the healthy community assessment tool. Intern. 2013;12:15.

105. McNeill C, Washburn L, Hadden KB, Moon Z. Evaluating the Effectiveness of the How to Talk to Your Doctor HANDbook Program. Health Lit Res Pract. 2019;3(2):e103-e9.

106. Mehrotra K, Chand P, Bandawar M, Rao Sagi M, Kaur S, G A, et al. Effectiveness of NIMHANS ECHO blended tele-mentoring model on Integrated Mental Health and Addiction for counsellors in rural and underserved districts of Chhattisgarh, India. Asian J Psychiatr. 2018;36:123-7.

107. Mifflin TM, Bzdell M. Development of a physiotherapy prioritization tool in the Baffin Region of Nunavut: a remote, under-serviced area in the Canadian Arctic. Rural Remote Health. 2010;10(2):1466-.

108. Mkwanazi NB, Rochat TJ, Bland RM. The Amagugu intervention: a qualitative investigation into maternal experiences and perspectives of a maternal HIV disclosure support intervention in rural South Africa. Health Policy Plan. 2017;32(9):1231-40.

109. Mocumbi S, McKee K, Munguambe K, Chiau R, Hogberg U, Hanson C, et al. Ready to deliver maternal and newborn care? Health providers' perceptions of their work context in rural Mozambique. Glob Health Action. 2018;11(1):1532631.

110. Modi D, Gopalan R, Shah S, Venkatraman S, Desai G, Desai S, et al. Development and formative evaluation of an innovative mHealth intervention for improving coverage of community-based maternal, newborn and child health services in rural areas of India. Glob Health Action. 2015;8:26769.

111. Mohammed A, Acheampong PR, Otupiri E, Osei FA, Larson-Reindorf R, Owusu-Dabo E. Mobile phone short message service (SMS) as a malaria control tool: a quasi-experimental study. BMC Public Health. 2019;19(1):1193.

112. Mohammed A, Acheampong PR, Otupiri E, Owusu-Dabo E. Symptom monitoring of childhood illnesses and referrals: A pilot study on the feasibility of a mobile phone-based system as a disease surveillance tool in a rural health district of Ghana. Health Inform J. 2019:1460458219879329.

113. Moore M, Roberts C, Newbury J, Crossley J. Am I getting an accurate picture: a tool to assess clinical handover in remote settings? BMC Med Educ. 2017;17(1):213.

114. Morgan D, Kosteniuk J, Seitz D, O'Connell ME, Kirk A, Stewart NJ, et al. A five-step approach for developing and implementing a Rural Primary Health Care Model for Dementia: a community-academic partnership. Prim Health Care Res Dev. 2019;20:11.

115. Mulholland P, O'Meara P, Walker J, Stirling C, Tourle V. Multidisciplinary practice in action: the rural paramedic -- it's not only lights and sirens. Journal of Emergency Primary Health Care. 2009;7(2):11p-p.

116. Mutale W, Stringer J, Chintu N, Chilengi R, Mwanamwenge MT, Kasese N, et al. Application of balanced scorecard in the evaluation of a complex health system intervention: 12 months post intervention findings from the BHOMA intervention: a cluster randomised trial in Zambia. PLoS ONE. 2014;9(4):e93977.

117. Nankervis K, Kenny A, Bish M. Enhancing scope of practice for the second level nurse: a change process to meet growing demand for rural health services. Contemporary Nurse: A Journal for the Australian Nursing Profession. 2008;29(2):159-73.

118. Ohl M, Dillon D, Moeckli J, Ono S, Waterbury N, Sissel J, et al. Mixed-Methods Evaluation of a Telehealth Collaborative Care Program for Persons with HIV Infection in a Rural Setting. J Gen Intern Med. 2013;28(9):1165-73.

119. Palmer RT, Biagioli FE, Mujcic J, Schneider BN, Spires L, Dodson LG. The feasibility and acceptability of administering a telemedicine objective structured clinical exam as a solution for providing equivalent education to remote and rural learners. Rural Remote Health. 2015;15(4):3399.

120. Pereira de Llano PM, Lange C, Pires Nunes D, Pastore CA, Pinto AH, Pilotto Casagranda L. Frailty in rural older adults: development of a care algorithm. Acta Paulista de Enfermagem. 2017;30(5):520-30.

121. Pesut B, Hooper B, Sawatzky R, Robinson CA, Bottorff JL, Dalhuisen M. Program Assessment Framework for a Rural Palliative Supportive Service. Palliative Care: Research & Treatment. 2013(7):7-17.

122. Petrie E. Action research informing the development of a conceptual model of care and service delivery to populations of interest in rural and remote communities. Australas. 2011;19:S102-S5.

123. Pimmer C, Mhango S, Mzumara A, Mbvundula F. Mobile instant messaging for rural community health workers: a case from Malawi. Glob Health Action. 2017;10(1):1368236.

124. Prashad AJ, Cameron BH, McConnell M, Rambaran M, Grierson LEM. An examination of Eyal & Hurst's (2008) framework for promoting retention in resource-poor settings through locally-relevant training: A case study for the University of Guyana Surgical Training Program. Can Med Educ J. 2017;8(2):e25-e36.

125. Prengaman M, Terry DR, Schmitz D, Baker E. The Nursing Community Apgar Questionnaire in rural Australia: An evidence based approach to recruiting and retaining nurses. Online J Rural Nurs Health Care. 2017;17(2):148-71.

126. Prengaman MP, Bigbee JL, Baker E, Schmitz DF. Development of the Nursing Community Apgar Questionnaire (NCAQ): a rural nurse recruitment and retention tool. Rural Remote Health. 2014;14:2633.

127. Purtill MA, Benedict K, Hernandez-Boussard T, Brundage SI, Kritayakirana K, Sherck JP, et al. Validation of a prehospital trauma triage tool: a 10-year perspective. Journal of Trauma. 2008;65(6):1253-7.

128. Raghavan M, Martin BD, Roberts D, Aoki F, MacKalski BA, Sandham JD. Increasing the enrolment of rural applicants to the faculty of medicine and addressing diversity by using a priority matrix approach to assign values to rural attributes. Rural Remote Health. 2011;11(2):1646.

129. Raghu A, Praveen D, Peiris D, Tarassenko L, Clifford G. Engineering a mobile health tool for resource-poor settings to assess and manage cardiovascular disease risk: SMARThealth study. BMC Med Inf Decis Mak. 2015;15:36.

130. Ray KN, Demirci JR, Bogen DL, Mehrotra A, Miller E. Optimizing Telehealth Strategies for Subspecialty Care: Recommendations from Rural Pediatricians. Telemed J E Health. 2015;21(8):622-9.

131. Rebello KE, Gosian J, Salow M, Sweeney P, Rudolph JL, Driver JA. The Rural PILL Program: A Postdischarge Telepharmacy Intervention for Rural Veterans. J Rural Health. 2017;33(3):332-9.

132. Reddy S, Orpin V, Herring S, Mackie-Schneider S, Struber J. Use of clinical guidelines in remote Australia: A realist evaluation. J Eval Clin Pract. 2018;24(1):152-8.

133. Reddy S, Wakerman J, Westhorp G, Herring S. Evaluating impact of clinical guidelines using a realist evaluation framework. J Eval Clin Pract. 2015;21(6):1114-20.

134. Reeve C, Humphreys J, Wakerman J. A comprehensive health service evaluation and monitoring framework. Eval Program Plann. 2015;53:91-8.

135. Reeve C, Humphreys J, Wakerman J, Carroll V, Carter M, O'Brien T, et al. Community participation in health service reform: the development of an innovative remote Aboriginal primary health-care service. Aust J Prim Health. 2015;21(4):409-16.

136. Riebschleger J, Norris D, Pierce B, Pond DL, Cummings C. Preparing Social Work Students for Rural Child Welfare Practice: Emerging Curriculum Competencies. Journal of Social Work Education. 2015;51:S209-S24.

137. Rieke JL, Safratowich M, Markland MJ. Discover Health Services Near You! The North Dakota Story: Part I. J Consumer Health Internet. 2009;13(2):143-55.

138. Riley MV, Vess J, Dumas BP. Home Therapy to Reduce Office Visits for Patients with Chronic Kidney Disease and Anemia. Nephrology Nursing Journal. 2017;44(1):29-34.

139. Rohatinsky NK, Jahner S. Supporting nurses' transition to rural healthcare environments through mentorship. Rural Remote Health. 2016;16(1):3637.

140. Sabatino V, Caramia MR, Curatola A, Vassallo F, Deidda A, Cinicola B, et al. Point-of-care ultrasound (POCUS) in a remote area of Sierra Leone: impact on patient management and training program for community health officers. J. 2020;09:09.

141. Sabesan S, Senko C, Schmidt A, Joshi A, Pandey R, Ryan CA, et al. Enhancing Chemotherapy Capabilities in Rural Hospitals: Implementation of a Telechemotherapy Model (QReCS) in North Queensland, Australia. J Oncol Pract. 2018;14(7):e429-e37.

142. Schuller KA, Cronin CE, Nicks SE, Jing X, Kingori C, Morrone M. Development and application of a rubric to compare strategies for improving access to health care in rural communities in the United States. Eval Program Plann. 2019;74:61-8.

143. Schuttner L, Sindano N, Theis M, Zue C, Joseph J, Chilengi R, et al. A mobile phone-based, community health worker program for referral, follow-up, and service outreach in rural Zambia: outcomes and overview. Telemed J E Health. 2014;20(8):721-8.

144. Stanley L, Min TH, Than HH, Stolbrink M, McGregor K, Chu C, et al. A tool to improve competence in the management of emergency patients by rural clinic health workers: a pilot assessment on the Thai-Myanmar border. Confl Health. 2015;9:11.

145. Tabatabaei-Moghaddam H, Sano Y, Mammen S. A case study in creating oral health messages for rural low-income families: a comparison to the cultural appropriateness framework. Health Promot Pract. 2014;15(5):646-53.

146. Terry DR, Baker E, Schmitz DF. Community assets and capabilities to recruit and retain GPs: the Community Apgar Questionnaire in rural Victoria. Rural Remote Health. 2016;16(4):3990.

147. Tetui M, Hurtig AK, Ekirpa-Kiracho E, Kiwanuka SN, Coe AB. Building a competent health manager at district level: a grounded theory study from Eastern Uganda. BMC Health Serv Res. 2016;16(1):665.

148. Yeboah-Antwi K, Snetro-Plewman G, Waltensperger KZ, Hamer DH, Kambikambi C, MacLeod W, et al. Measuring teamwork and taskwork of community-based "teams" delivering life-saving health interventions in rural Zambia: a qualitative study. BMC Med Res Methodol. 2013;13:84.

149. Yonge O, Myrick F, Ferguson L. The process of developing a framework to guide rural nurse preceptors in the evaluation of student performance. Nurse Educ Pract. 2011;11(2):76-80.
